# Effects of different culture media on physiological features and laboratory scale production cost of *Dunaliella salina*

**DOI:** 10.1016/j.btre.2020.e00508

**Published:** 2020-07-24

**Authors:** Guilherme Augusto Colusse, Carlos Rafael Borges Mendes, Maria Eugênia Rabello Duarte, Julio Cesar de Carvalho, Miguel Daniel Noseda

**Affiliations:** a Postgraduate Program in Bioprocess Engineering and Biotechnology, Federal University of Paraná, Av. Cel. Francisco H. dos Santos, 100, CEP 81531-990, PO BOX 19011, Curitiba, Brazil; b Biochemistry and Molecular Biology Department, Federal University of Paraná, Av. Cel. Francisco H. dos Santos, 100, CEP 81531-980, PO BOX 19046, Curitiba, Brazil; c Institute of Oceanography, Federal University of Rio Grande, Av. Itália, Km 8, CEP 96203-900, Rio Grande, Brazil; d Bioprocess Engineering and Biotechnology Department, Federal University of Paraná, Av. Cel. Francisco H. dos Santos, 100, CEP 81531-990, PO BOX 19011, Curitiba, Brazil

**Keywords:** Microalgae, Vegetative phase, Economic analysis, Photosynthetic pigments, Culture medium, Biomass production

## Abstract

Microalgal cultivation system depends on optimal parameters obtained from laboratory conditions to overcome high-value compounds production and decrease cost. In this study, the laboratory-scale cultivation of *Dunaliella salina* was performed to explore the cost and biochemical effects on biomass cultivated using F/2, Conway, and Johnson media. *D. salina* cultivation was monitored by cell counting, dry biomass measurements and biochemical analyses. Photosynthetic pigment profiles were identified and quantified through high-performance liquid chromatography (HPLC) whereas economic evaluation was based on media cost. Cellular growth parameters were significantly improved by Conway medium, whereas maximum accumulation of pigments, especially violaxanthin (0.855 mg g^−1^) and chlorophyll *a* (14.255 mg g^−1^) were observed when Johnson medium was used. Conway-biomass showed 43 % of total lipid content. This value represents 1.5 and 1.4 times higher than lipid content found in Johnson- and F/2-biomass, respectively. Furthermore, media cost presented a variation of US$1.17–49.62 for each 10^3^ L of media mainly due to NaNO_3_, KNO_3,_ and cyanocobalamin supplementation. Thus, biomass production showed a cost variation of US$4.64–301.61 per kg on a dry weight basis. These outcomes emphasize the suitability of laboratory parameters and cost comparison among biomass produced by different media.

## Highlights

• Comparison among well-known marine microalgae culture media was performed.
• Conway medium increased cellular growth of *Dunaliella salina*.
• Johnson medium improved accumulation of photosynthetic pigments.
• Microalgal biomass showed a cost variation of US$4.64–301.61 per kg in lab scale.
• Monitoring of pigment profile proved to be essential in microalgal parameters.

## Graphical abstract

**Figure:**
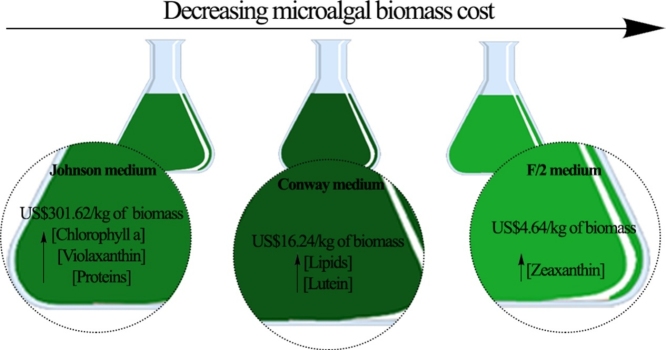

## 1 Introduction

The unicellular green halophilic flagellate microalga, *Dunaliella salina*, is a well-organized cell factory and the most feasible source of natural β-carotene worldwide. Scientific knowledge about *D. salina* cultivation is crucial to determine how to increase biomass production at low cost, intensifying industrial production. Several studies have reported the importance of looking-forward technologies that increase microalgal biomass and pigment production in a cost-effectively economic scenario [[1], [2], [3]].

Even though *D. salina* cultivation can be a potential source of high-added value pigments, laboratory parameters and media costs are concerns to be overcome. Previous studies have shown that different microalgal medium and culture conditions can affect the cellular metabolism directly and therefore the production cost [[4], [5], [6]]. Microalgal typical medium can cost from US$ 2.6 to 18.3 each 10^3^ L in laboratory-scale cultivation systems, and medium components such as NaNO_3_ and K_2_HPO_4_ can represent 83.3 and 31 % respectively, in the medium cost [6]. Moreover, medium component replacement by economical sources can provide higher biomass production as well as an increase on protein, lipid and docosahexaenoic acid (DHA) content [7].

Furthermore, culture conditions can interfere directly with the microalgal metabolism, affecting the production of molecules such as pigments. Some studies have addressed significant effects of light and adaptive evolution [8], temperature [9] and gene expression responses to stress [10] on pigment accumulation from *D. salina* cells. In addition to cultivation features, strategies to enhance carotenoid biosynthesis using leaf extracts was described by Einali and colleagues (2017) [11]. However, few studies have demonstrated the correlation of microalgal biomass cultivated under common media [6,7,12], pigment profile and laboratory cost in *D. salina* cultivation (no stress applied).

In this study, we evaluated the performance of different microalgal culture media on *D. salina* cultivation and analyzed their effects on biomass composition and laboratory cost production. The study evaluated crucial parameters related to microalgae cultivation: (i) cellular growth and physicochemical parameters, (ii) pigment profile in different media, and (iii) parameterizing cost under laboratory-scale production. The results of this study may provide useful information about the enhancement of microalgal biomass production related to the chosen medium and laboratory-scale production cost.

## 2 Material and methods

**Fig. 1:**
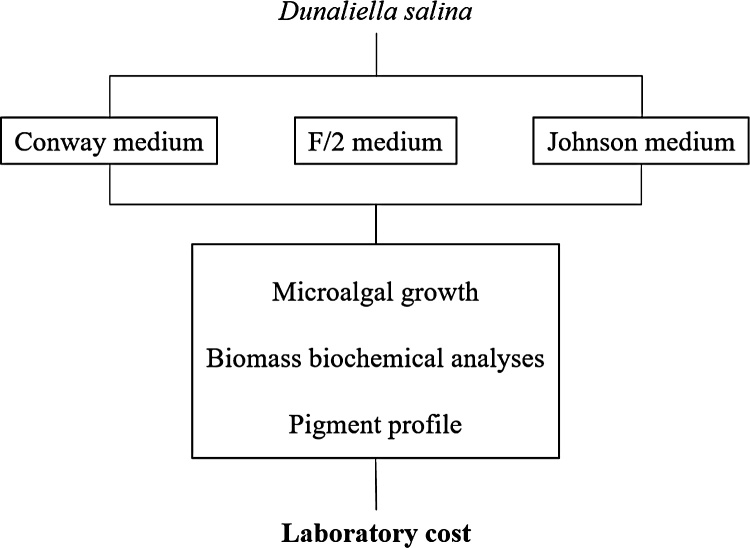
Schematic step-by-step approach of this study.

In order to obtain a complete picture of the methodology used in this study, we presented a step-by-step approach as shown in Fig. 1.

### 2.1 *Dunaliella salina* strain and culture maintenance

*D. salina* strain (BMAK 116) was obtained from the Aidar & Kutner Collection Bank (BMA&K, Oceanographic Institute at the São Paulo University, Brazil). The strain was maintained photoautotrophically in Conway medium [13], using artificial seawater (35 g L^−1^, Salinity® salt). To proceed with the experimental design, the inoculum was grown in a two-liter flask (10 % v/v inoculation, initial concentration of 30 × 10^4^ cells mL^−1^) in the same medium at 23 ± 1 °C under 12:12 light/dark cycle (100 μmol m^-2^ s^−1^) for 5 days.

### 2.2 Experimental design

The microalga was cultivated in three common media described in the literature. Conway, F/2 [14], and Johnson [15] media were prepared individually into two-liter flasks with a working volume of 1600 mL using artificial seawater (35 g L^−1^, Salinity® salt). pH was adjusted to 7.5, and the flasks were autoclaved. Inoculation was done based on 10 % v/v of stock culture from previous step after centrifugation (851 x g for 15 min) to avoid nutrient interferences on media (initial concentration of 16 × 10^4^ cells mL^-1^ after inoculation). The experiment was carried out during 15 days under 12:12 light/dark cycle with cool white fluorescent lamps providing 100 μmol m^-2^ s^−1^. During all cultivation period, continuous sterilized compressed air was provided with a flow rate of 2 L min^−1^ (without supplementary CO_2_). All components used were analytical grade.

### 2.3 Microalgal growth and offline measurement

Microalgal culture samples were taken every 24 h. Dry biomass quantification was determined by filtration using a pre-weighted Macherey-Nagel GF-1 glass-fiber filter and dried at 80 °C until constant weight. Biomass productivity (in terms of g L^−1^ per day) was calculated using Eq. (1).

(1) $$\text{Productivity=}\frac{\text{(}\text{X}\text{f}\text{-X}\text{i}\text{)}}{\text{(}\text{t}\text{f}\text{-t}\text{i}\text{)}}$$

Where *X_f_* and *X_i_* correspond to final and initial biomass (g L^−1^), respectively related to final (*t_f_*) and initial time (*t_i_*). Cell counting was performed by optical microscopy using a Neubauer hemocytometer for cell growth analysis. Microalgae growth performance was measured by the following Eqs. (2), (3), (4), (5) according to Guillard (1973) [16] and Wood et al. (2005) [17]:

(2) $$\text{Growth rate }\left(\text{r}\right)\text{=}\frac{\text{ln}\text{(}\text{N}\text{f}\text{/N}\text{i}\text{)}}{\text{t}\text{f}\text{-t}\text{i}}$$

(3) $$\text{Doubling time }\left(\text{T2}\right)\text{=}\frac{\text{0.6931 }}{\text{r}}$$

(4) $$\text{Division per day }\left(\text{k}\right)\text{=}\frac{\text{r}}{\text{0.6931}}$$

(5) $$\text{Maximum cellular yield }\left(\text{R}\right)\text{=}\;\text{N}\text{f}\text{-N}\text{i}\;$$

Where *N_f_* and *N_i_* correspond to final and initial cell density, respectively related to their specific final (*t_f_*) and initial time (*t_i_*) in days. pH and conductivity were measured to monitor variation in the culture during the whole experiment.

### 2.4 Photosynthetic pigment profile and quantification

All analyses were done on a daily basis. Briefly, a culture volume of 5 mL was filtered using a Macherey-Nagel GF-1 glass-fiber filter (47 mm, 0.7 μm). The biomass-filter was resuspended in an acetone-water mixture (4 mL, 90 % v/v) and shaken vigorously. The tubes were maintained in the dark at 4 °C for 20 h to extract all the photosynthetic pigments. The supernatant was recovered through centrifugation (1915 x g for 10 min), and the optical absorbance was determined using a spectrophotometer BEL SP1105 at 480, 510, 664 and 750 nm. Chlorophyll_a_ (Chl), pheophytin_a_ (Phe) and total carotenoids (Car) were calculated according to spectrophotometric equations described in the literature [18,19]. The analysis was performed in triplicate and the results were shown in mean ± SD (Standard Deviation).

Biomass was also centrifuged at the end of the experiment and lyophilized for pigment profile analyses. Moreover, biomass mix of all 3 replicates was analyzed using high-performance liquid chromatography (HPLC). Freeze-dried samples of 0.0042–0.0368 g were placed in a screw-cap tube with 6 mL of 95 % cold-buffered methanol (2% ammonium acetate) containing 0.05 mg L^−1^ trans-β-apo-8’-carotenal (Fluka, Buchs, Switzerland) as internal standard. The samples were sonicated for 5 min in an ice-water bath, placed at −20 °C for 1 h and centrifuged at 1100 g for 5 min at 3 °C. Supernatants were filtered through Fluoropore PTFE membrane filters (0.2-μm pore size; Merck Millipore Ltd, Billerica, MA, USA) to remove cell debris. Immediately before injection, 1 mL of sample was mixed with 400 mL of Milli-Q water in 2-mL amber glass sample vials, and vials were placed in the HPLC cooling rack (4 °C). Method procedures for HPLC analyses (using a monomeric C8 column with a pyridine-containing mobile phase) are fully described in Zapata et al. (2000) [20]. The detection limit and quantification procedure of this method were done according to Mendes et al. (2007) [21]. Pigments were identified from both absorbance spectra and retention times from the signals in the photodiode array detector (SPDM20A) (Shimadzu Corporation, Kyoto, Japan). Peaks were integrated using LC-Solution software, but all peak integrations were checked manually and corrected where necessary. The HPLC system was previously calibrated with pigment standards from DHI (Institute for Water and Environment, Denmark). For correction losses and volume changes, the concentrations of the pigments were normalized to the internal standard.

### 2.5 Biomass biochemical analyses

Biomass were centrifuged and lyophilized for biochemical analyses. The spectrophotometric method of Lowry et al. (1951) [22] was used to determine protein content in the dried biomass. Total carbohydrates were determined by the phenol-sulfuric methodology [23] and quantified spectrophotometrically using a standard glucose curve (490 nm), while total lipids were determined by the gravimetric method described by Blight and Dyer (1959) [24]. The analysis was performed in triplicate and the results were shown in mean ± SD (Standard Deviation).

### 2.6 Laboratory economic analysis

Medium component costs were obtained by several quotations from laboratory companies and chosen based on the minimum price. The contribution of microalgal media was evaluated based on the total media cost, individual media component cost, and indoor biomass production cost. The individual component cost was expressed as a percentage of the total medium cost. To evaluate laboratory pigment costs, the analysis was performed based on pigment content and dry biomass cost. The individual pigment cost was expressed in US$ dollar and 1 kg basis of powdered pigment.

### 2.7 Statistical analysis

Each experiment was performed in triplicate, and the results were shown in mean ± SD (Standard Deviation). Results were analyzed by ANOVA at 95 % confidence interval and Tukey’s test with significance at *p* < .05.

## 3 Results and discussion

### 3.1 *Dunaliella salina* culture kinetics, pH and conductivity

**Fig. 2:**
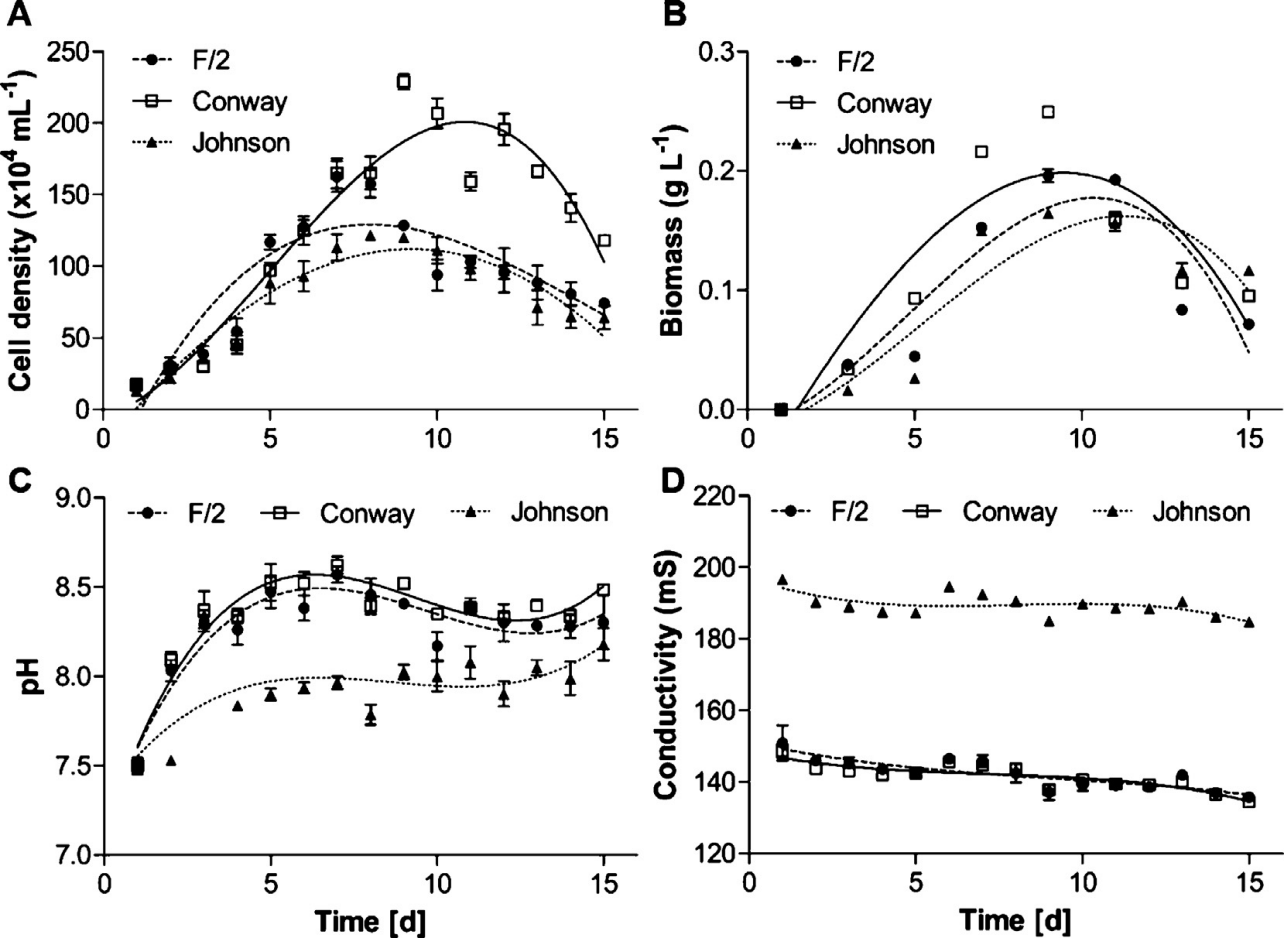
Characterization of growth behavior (cell density and biomass), pH and conductivity profiles of *D. salina* cultivated in F/2, Conway and Johnson media.

Differences in growth characteristics of *D. salina* during 15 days cultivation in microalgal common culture media in batch-culture mode are shown in Fig. 2. A total of 229 ± 5.29 × 10^4^ mL^−1^ cells was achieved when Conway medium was used in the cultivation. The highest cell density in the Conway medium was 1.7 and 1.9 times higher than cells cultivated in F/2 and Johnson media in the same period, respectively (Fig. 2A). Thus, F/2 and Johnson media presented 162 ± 10.69 and 121 ± 3.05 × 10^4^ mL^−1^ cells, respectively. No cellular morphology differences were observed during cultivation in the different media.

Biomass production followed the same pattern as cellular growth (Fig. 2B). Biomass was highest in Conway medium with 0.25 g L^−1^ followed by 0.19 g L^−1^ and 0.16 g L^−1^ for F/2 and Johnson media, respectively. Higher cellular concentrations are reported in *D. salina* and *D. tertiolecta* cultivation due to the presence of some additives in culture media such as pentoses [25] and growth hormone [26] presenting a biomass production of 1.25 and 0.8 g L^−1^ respectively. In comparison with other studies on microalgal medium evaluation, Nahidian and colleagues (2018) [12] highlighted possible improvements on microalgae cultivation due to microalgal dependency to the medium nutrient content. Moreover, to increase *D. salina* production, Sathasivam and Juntawong (2013) [27] presented a modified Johnson medium with altered MgSO_4_ and NaHCO_3_ concentrations, addition of NaVO_3_ and elimination of MgCl_2_. The authors reported a production of 508 × 10^4^ cells mL^−1^. However, the present study exhibited characteristics related to the use of common media without any supplementation on laboratory scale.

According to Fig. 2C, pH showed an increase in all media. Similar pH increase was reported and it might be favorable for the induction of carotenoids [28] and biomass production [29]. Conductivity (Fig. 2D) showed a decrease of 10, 9.4 and 6 % in F/2, Conway and Johnson media, respectively compared to the initial values. These results can be related to nutrient consumption by the cells over the cultivation period. Conductivity analysis is an useful approach in microalgae cultivation since it can be used to monitor nutrient availability [30] and microalgal dewatering performance [31].

Regarding *D. salina* kinetics, cellular growth parameters were measured to evaluate the media effects on cell metabolism (Table 1). Conway medium proved to have a significant positive impact on *D. salina* growth parameters when compared to biomass produced using F/2 and Johnson media.

**Table 1:** Growth and physicochemical parameters for *Dunaliella salina* cultivation in different culture media at the end of experiment*.

Growth parameters			
	F/2	Conway	Johnson
Maximum cellular density (x10^4^ cells mL^−1^)	162 ± 10.69^b^	229 ± 5.29^a^	121 ± 3.05^c^
Division per day (k)	0.53	0.54	0.51
Doubling time (T_2_)	1.85	1.84	1.94
Growth rate (r)	0.373	0.375	0.356
Maximum cellular yield (R) (x10^4^ cells mL^−1^)	145 ± 9.71^b^	211 ± 3.05^a^	108 ± 3.78^c^
Dry biomass (g L^−1^)	0.19 ± 0.005^b^	0.25 ± 0.002^a^	0.16 ± 0.001^c^
Productivity (g L^−1^ d^−1^)	0.024	0.031	0.020
pH	8.30 ± 0.15	8.48 ± 0.02	8.18 ± 0.08
Conductivity (mS cm^−1^)	135.76 ± 0.35^b^	134.46 ± 0.85^b^	184.83 ± 0.65^a^

* Averages followed by the same letter do not significantly differ (*p* <  .05).

Productivity affects the cost of microalgal biomass and molecules of interest. As can be seen further on this study, parameters such as division per day, doubling time, growth rate, dry biomass and biomass pigment composition from the evaluated media can affect directly the laboratory production cost. According to Chavoshi and Shariati, (2019) [32], decrease on cell division and biomass production are related to cellular self-shading and photoinhibition observed in autotrophic cultures. Besides medium nutrients, variables such as quality and quantity of light also affect biomass productivity rate [33].

KNO_3_ and NaHCO_3_ increase growth parameters in *D. salina* cultivation as previously reported [34]. These authors also evaluated microalgae cultivation by adopting an aeration rate of 0.5 L min^−1^ and an irradiance of 70 μmol photons m^-2^ s^−1^. However, for this study we used an aeration rate of 2 L min^−1^ and 100 μmol photons m^-2^ s^−1^ in all cultivation steps.

Several aspects concerning microalgal development parameters are essential to understand dynamic kinetic growth, and can be evaluated through model equations as described by Fachet and colleagues (2014) [35] or to choose a specific culture medium related to the production of a molecule of interest. In this sense, the Conway medium provided the most suitable nutrient conditions for the growth of *D. salina* in vegetative stage cultivation. These outcomes indicate that further evaluation related to biomass enhancement and growth parameters with Conway medium would be interesting since it has presented better results than other culture media evaluated in laboratory scale from this study.

### 3.2 *D. salina* pigment quantification during cultivation

**Fig. 3:**
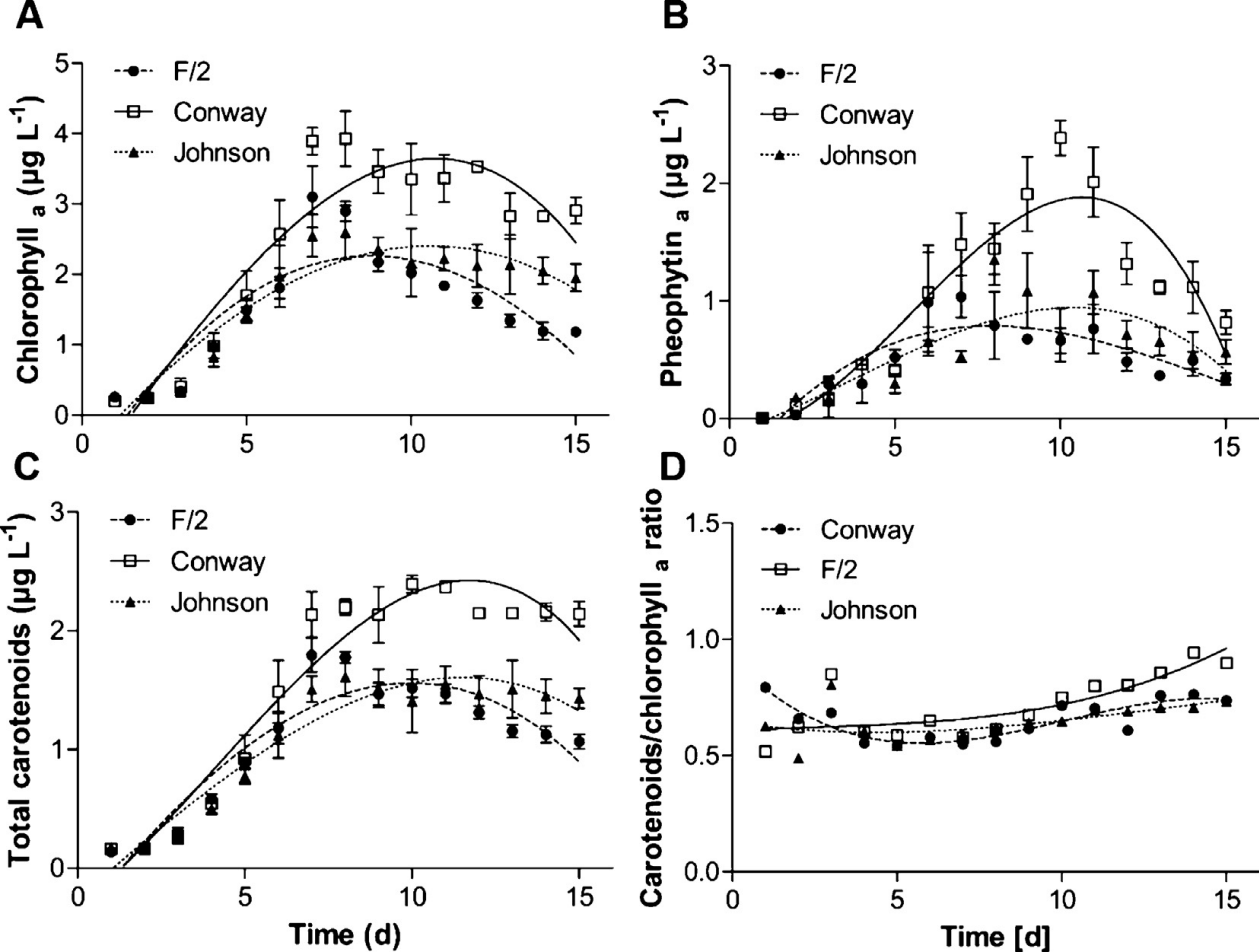
*D. salina* photosynthetic pigments profile, obtained by spectrophotometry, over cultivation period using F/2, Conway, and Johnson media.

In order to evaluate the pigment profile during cultivation, photosynthetic pigments were monitored once a day using spectrophotometric analysis (Fig. 3). Pigments concentrations are shown in terms of wet basis (w/v). We found that the increase in cell concentration (previous step) leads to an increase in pigment content per cell, in particular chlorophyll *a* and pheophytin *a*.

Conway medium showed the highest chlorophyll *a* content (3.92 ± 0.43 μg L^−1^) at day 7 of cultivation as presented in Fig. 3A. It corresponds to an increase of 21 and 34 % when compared with chlorophyll *a* content from F/2- and Johnson-biomass, respectively. According to Fig. 3A and 3B, F/2-biomass presented chlorophyll *a* and pheophytin *a* contents of 3.10 ± 0.43 and 1.03 ± 0.17 μg L^−1^, respectively, reaching the maximum pigment production at 7 days of cultivation. Furthermore, Johnson-biomass showed the maximum chlorophyll *a* and pheophytin *a* production at the 8th day of cultivation with 2.59 ± 0.37 and 1.35 ± 0.21 μg L^−1^, respectively.

Moreover, several aspects on microalgal cultivation can increase (or decrease) pigment content. According to Wu and colleagues (2016) [9], temperature and light intensity showed positive impact in *D. salina* pigment accumulation, mainly β-carotene. These authors also discussed the interference of other factors such as nutrients availability (KNO_3_, CO(NH_2_)_2_ and NaHCO_3_) and strain used. Furthermore, light emitting diodes (LED) can also influence pigment production in *D. salina* cultivation [[36], [37]].

Total carotenoids/chlorophyll *a* ratio is an indicator of stress in microalgal cultivation related to exposure to several biotic and abiotic factors. In this study, non-stressed microalgal cells present total carotenoid (Fig. 3C) and chlorophyll *a* content at the same level during the cultivation period, as can be seen in Fig. 3D. Cultivation variables such as light and nitrogen deprivation can induce carotenoid synthesis in microalgal cells [38] and can be monitored by the total carotenoids/chlorophyll *a* ratio [37].

### 3.3 *D. salina* pigment profile at the end of the cultivation period

The effect of microalgal media on *D. salina* pigment profile was measured by HPLC at the end of microalga culture (Table 2**)**. The usual approach to analyze pigment content from microalgal biomass is by colorimetric assays, however HPLC allows the identification and quantification of a wider range of pigments, and was used to gather information about pigment profile and consequent influences from culture media variables [39].

**Table 2:** HPLC pigments (chlorophylls and carotenoids) quantification (mg g^−1^ of dry biomass) of *D. salina* at the end of the cultivation period*.

Pigments (mg g^−1^)			
	F/2	Conway	Johnson
Neoxanthin	0.497	0.668	0.671
Violaxanthin	0.454	0.568	0.855
Zeaxanthin	0.159	0.115	0.007
Lutein	2.123	2.596	1.252
Chlorophyll *b*	3.284	4.247	5.974
Chlorophyll *a*	8.304	10.267	14.255
β-carotene	n/d**	n/d	n/d

* Biomass mix of all 3 replicates.

** n/d: not detected.

We found that biomass obtained from Johnson medium presented the highest neoxanthin (0.671 mg g^−1^), violaxanthin (0.855 mg g^−1^), chlorophyll *a* (14.255 mg g^−1^) and chlorophyll *b* (5.974 mg g^−1^) content when compared with biomass from Conway and F/2 media. Furthermore, chlorophyll *a* was highly enriched in biomass produced in Johnson medium in a dry basis (w/w), differing from the previous section, in which Conway medium proved to increase this molecule during cultivation. It suggests that Johnson medium enhances chlorophyll *a* inside the cell, and no longer to cellular growth. Differences on pigment profile related to different nitrate concentration were reported by Gao and coworkers (2016) [39]. These authors showed an intrinsic relationship among photosynthetic pigments such as chlorophyll *a*, β-carotene, vaucheriaxanthin-ester and violaxanthin, from *Vischeria stellata*, and the nitrate concentration.

According to the results achieved in the present study, Conway medium induced lutein synthesis (2.596 mg g^−1^). This pigment showed an increase of 18 and 51 % compared to lutein concentration from F/2- and Johnson-biomass, respectively. Microalgal pigment variation is reported in literature. Srinivasan and colleagues (2018) [5] observed an increase in lutein and β-carotene content in *D. salina* cultivation under nutrient deficit conditions and sodium bicarbonate addition (100 mM). It reduced oxidative stress, lowered lipid peroxidation damage and improved antioxidant enzyme activities. Taking advantage of the fact that culture medium interferes in the microalgal pigment profile, we reasoned that it is an affordable and feasible approach to enhance specific pigment in a large scale microalgal facility.

Pigment profile plays an important role in microalgal species characterization as well as on the evaluation of cultivation features in microalgae production. Furthermore, medium components can be supplemented in order to increase certain pigment of interest since our experimental results have demonstrated that each medium has different impact on pigment production.

### 3.4 *D. salina* biomass biochemical composition

*D. salina* cells were grown for up to 15 d in different culture media. Biochemical parameters, such as lipids, proteins and carbohydrates were monitored in the biomass at the end of the cultivation period. Several studies reported that there is a change from photosynthetic carbon flow to energy-rich chemical compounds such as lipids, proteins and carbohydrates during cultivation [40,41]. These molecules act as storage components in the cellular mechanism and can be influenced by diverse cultivation features [[42], [43], [44]]. In the present study, *D. salina* biomass produced in different culture media showed significant differences on biochemical composition as can be seen in Table 3.

**Table 3:** Effect of culture media on lipid, protein, and carbohydrate content from *D. salina* biomass*.

Parameters			
		Culture media	
	F/2	Conway	Johnson
Lipids (%)	31.3 ± 1.15^b^	43.3 ± 2.3^a^	28.6 ± 1.15^b^
Proteins (%)	19.1 ± 1.96^b^	23.4 ± 0.89^b^	38.3 ± 2.88^a^
Carbohydrates (%)	5.6 ± 0.28^b^	6.9 ± 0.13^b^	11.3 ± 0.45^a^

* Averages followed by the same letter do not significantly differ (*p* <  .05).

Biomass produced using Conway medium showed a 43.3 % of lipid content. This value represents 1.5 and 1.4 times higher than lipid content obtained in Johnson- and F/2 biomass, respectively. According to Ahmed and colleagues (2017) [4], lipid enhancement is strictly dependent on salt addition to the culture medium in *D. salina* cultivation. The authors recorded a 22.28 % of total lipid content at optimum salinity level (2 M NaCl). In the present study we focused on microalgal effects by medium components; therefore, synthetic seawater (with artificial sea salt) was used equally in all media preparation and the salt effect was not analyzed.

Microalgal biomass obtained using Johnson medium presented the highest protein content, differing significantly from protein content obtained in F/2- and Conway biomass. It showed a 38.3 % protein content, followed by 23.4 and 19.1 % obtained from Conway and F/2 biomass, respectively. High-quality protein production potential from *D. salina* was reported by Sui et al. (2019) [45]. The authors reported that growth phase and light regime also affect protein content and essential amino acid levels on *D. salina* cultivation. These results highlight the importance of studies that correlate biomass biochemical composition with microalgae cultivation features.

Carbohydrate content showed 11.3 % in biomass obtained using Johnson medium. It presented a production of 1.6 and 2 times higher than carbohydrate content found in biomass produced by Conway and F/2 media. de Freitas and coworkers (2019) [25] presented a carbohydrate content of 20.5 % in *D. salina* cultivation. The authors studied the positive effects of d-xylose and l-arabinose addition into the culture media related to carbohydrate and protein content, and biomass production (1.38 g L^−1^). Besides structural features in the microalgal cell, carbohydrates derived from microalgae have also received attention due to its biological application in medical and pharmaceutical areas such as antitumor properties [46], antitussive and bronchodilatory activity [47], plant growth biostimulant action [48], and antimicrobial property [49].

Different biochemical contents were also reported for microalgal biomass cultivated under different media [6,12,50]. The biochemical comparison among microalgal biomass suggests that medium components present an intrinsic relationship in cell metabolism promoting the increase of a specific molecule; therefore, it can be further used to promote the enhancement of biocompounds of interest.

### 3.5 Economic evaluation

To further compare culture media, we performed an economic analysis of each medium (Table 4). The basis considered for calculation was 10^3^ L of culture medium, in batch-culture mode, and the cost to produce 1 kg of dry biomass.

**Table 4:** Media economical evaluation (in US$ dollars)*.

Cost			
	F/2	Conway	Johnson
10^3^L medium	1.17	4.33	49.62
kg of dry biomass	4.64	16.24	301.62

* Average exchange rate (February 2020) US$1.00 = R$4.31.

Based on the economic analysis of the media in laboratory scale, F/2 medium showed a contribution to biomass cost of US$4.64/kg whereas Conway medium presented a cost of US$16.24/kg. For Johnson media, which had the highest cost not only for medium components but also for dry biomass, showed a production cost of US$49.62 and US$301.62, respectively. In our previous study, culture media showed a cost from US$ 2.6 to 18.3 for 10^3^ L and dry biomass production cost from US$6.2 to 40.3/kg [6].

**Fig. 4:**
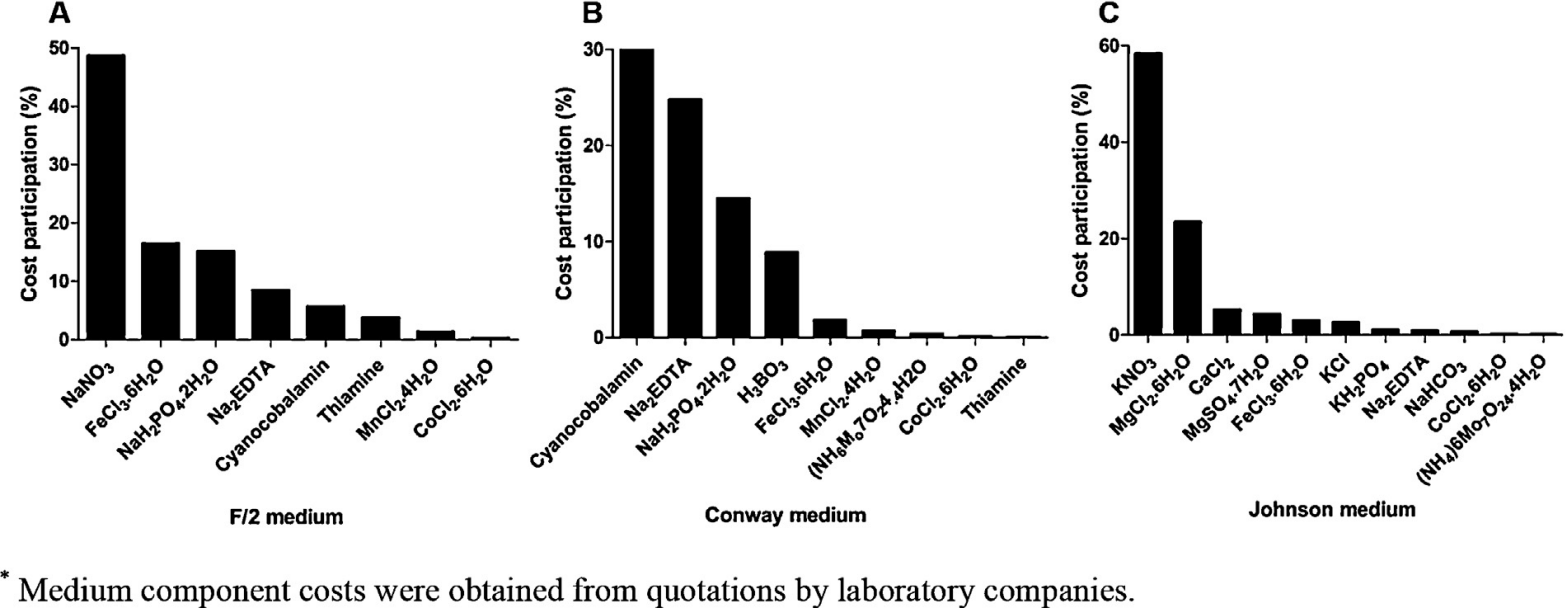
Component participation in the media cost of F/2, Conway and Johnson media*. *Medium component costs were obtained from quotations by laboratory companies.

Moreover, Li and colleagues (2011) [51] showed in an economic analysis of *Haematococcus pluvialis* cultivation, that the proposed biomass production cost was around US$18/kg in a dry weight basis. Interestingly, media cost variation is dependent of several parameters and play an important role on microalgal production process. Besides energy consumption and harvesting methods [52], nutritional components significantly contribute to final medium cost. Fig. 4 presents the components with major cost participation in the tested media. Components that presented <0.1% of cost participation were not listed.

The components with the highest cost participation were NaNO_3_ (48.6 %), cyanocobalamin (30 %) and KNO_3_ (58.3 %) at the F/2 (Fig. 4A), Conway (Fig. 4B) and Johnson (Fig. 4C) media composition, respectively. MgCl_2_.6H_2_O and KNO_3_ content from Johnson medium represents a total of 81 % of total cost while cyanocobalamin, Na_2_EDTA, NaH_2_PO_4_.2H_2_O and H_3_BO_3_ content in Conway medium and NaNO_3_, FeCl_3_.6H_2_O and NaH_2_PO_4_.2H_2_O content in F/2 medium show a 78 % and 80 %, respectively, of participation on total cost in these media.

It is worth mentioning that some medium components could be easily replaced by low/null-cost effluents from the agribusiness industry. The use of low-cost nutrients or residues such as sugarcane vinasse [53] or anaerobic digestate [54] in microalgae cultivation could provide benefits to the process besides decreasing the medium cost. Wang and colleagues (2018) [55] presented an increase on *Chlorella pyrenoidosa* productivity of 4.76 times than that cultured at BG-11 medium using tofu whey wastewater as basal medium.

In order to make the presented study applicable for industrial purposes, extended evaluations of low-cost nutrient addition and cultivation features such as CO_2_ injection and light intensity studies would be required. However, detailed results from medium components participation presented here are essential to support information to be elucidated in upcoming studies.

Furthermore, several studies have examined different ways to decrease microalgal medium cost [[55], [56], [57]], but few focused on pigment production and cost related with laboratory scale basis. In this sense, this study presented information of pigment cost based on the use of common media described in the literature, in their original concentration. To assess a relationship that may correlate biomass and pigment, estimations of pigment cost related to biomass production were taken to assess different economic effects resulting from microalgal media. Table 5 presents the estimation of pigment cost production derived from F/2, Conway and Johnson biomass in 1 kg basis of powdered pigment.

**Table 5:** Estimated pigment economical evaluation based on laboratory production scale (1 kg basis of powdered pigment, US$ dollars).

Pigments			
	F/2	Conway	Johnson
Neoxanthin	9,336.01	24,311.37	449,508.19
Violaxanthin	10,220.26	28,591.54	352,771.92
Lutein	2,185.58	6,255.77	240,910.54
Chlorophyll *b*	1,412.91	3,823.87	50,488.78
Chlorophyll *a*	558.76	1,581.76	21,158.89

In all biomass produced, pigment cost was excessively high. Johnson medium components increase pigment cost in all pigments analyzed compared with pigments extracted from F/2 and Conway biomass. The increase of pigment cost is related to biomass production and media component participation in the media. Chlorophyll *a* presented the lower cost among all pigment comparison since it was the most abundant molecule showed in all biomass (see Table 2).

Due to the toxic effects of synthetic pigments, there is an increase on the use of natural colorants obtained from microalgae in several promising applications such as diagnostics, biomedical research, dairy products, therapeutics, and colorings in cosmetics [58].

Studies showed that pigments play an important role mainly in biomedical areas. Brazionis and coworkers (2008) [59] suggested that pigments such as lutein can protect against diabetic retinopathy. Furthermore, neoxanthin was described to reduce cell viability on prostate cancer cell lines [60] and violaxanthin presented antiproliferative activity on human mammary cancer cell line [61]. In addition, chlorophyll is used as natural color in food, cosmetic and pharmaceutical products with high-added value [58].

In most microalgal mass cultures, particularly those of outdoor raceway ponds, pigments production as well as other molecules of interest have been studied with the aim of enhancing productivity and decreasing their costs [62]. Li and colleagues (2011) [51] presented an economic evaluation of an industrial plant with a total capacity of 900 kg of natural astaxanthin per year. The authors showed a total cost of US$718/kg of powered astaxanthin using the green biflagellate microalga *Haematococcus pluvialis* as a natural producer.

The addition of low-cost components such as ammonium sulfate, calcium superphosphate and urea can decrease medium cost and raise pigment content per unit of biomass [62]. It confirms that studies related to alternative cost-effective culture media are essential to enhance growth and pigment content in addition to reduce cost production. Moreover, information about laboratory-scale production is essential since it can be used as basic science to be part of the decision-making process and to overcome challenges faced in a large scale microalgal facility.

## 4 Conclusions

In our study, cultivating *D. salina* cells using F/2, Conway, and Johnson media showed several differences in growth, biochemical characteristics, pigment profile, and cost. Protein content (38.3 %) and total carbohydrate (11.3 %) were greatly improved in Johnson-biomass, whereas Conway-biomass showed higher lipid production (43.4 %). Moreover, Johnson-biomass showed a 14.255 mg g^−1^ of chlorophyll *a* content, while Conway medium presented a 2.596 mg g^−1^ of lutein production besides allowed faster cell growth.

Media cost showed a variation of US$1.17–49.62 each 10^3^ L and components such as NaNO_3_, cyanocobalamin and KNO_3_ represented 48.6, 30.0 and 58.3 %, respectively in final medium cost participation. Experimentally, we verified that pigment laboratory costs, although unfeasible in a large production scale based on the results found in this study (US$558.76–449,508.19, 1 kg basis of powdered pigment), presented enormous differences in terms of production only by changing the culture media.

Additionally, to further evaluate the effects of culture media on *D. salina* cultivation, future research might involve medium optimization based on the use of low-cost-added supplements related to the production of pigments as well as in-depth studies of scale-up outdoor experimental tests. Also, the increase of carbon dioxide to *D. salina* cultivation may yield greater biomass production and improvements for future prospects of this topic.

## Funding

This work was supported by the Conselho Nacional de Desenvolvimento Científico e Tecnológico (CNPq) – Brazil [Universal Call grant number: 462414/2014-0]; and Coordenação de Aperfeiçoamento de Pessoal de Nível Superior (CAPES) – Brazil (Finance Code 001).

## CRediT authorship contribution statement

**Guilherme Augusto Colusse:** Investigation, Formal analysis, Writing - original draft. **Carlos Rafael Borges Mendes:** Investigation, Writing - review & editing. **Maria Eugênia Rabello Duarte:** Supervision, Conceptualization. **Julio Cesar de Carvalho:** Supervision, Data curation, Methodology. **Miguel Daniel Noseda:** Conceptualization, Writing - review & editing, Supervision, Project administration.

## Declaration of Competing Interest

The authors declare that they have no known competing financial interests or personal relationships that could have appeared to influence the work reported in this paper.
